# Mitochondrial DNA Promotes NLRP3 Inflammasome Activation and Contributes to Endothelial Dysfunction and Inflammation in Type 1 Diabetes

**DOI:** 10.3389/fphys.2019.01557

**Published:** 2020-01-17

**Authors:** Camila A. Pereira, Daniela Carlos, Nathanne S. Ferreira, Josiane F. Silva, Camila Z. Zanotto, Dario S. Zamboni, Valéria D. Garcia, Dora Fix Ventura, João S. Silva, Rita C. Tostes

**Affiliations:** ^1^Department of Pharmacology, Ribeirão Preto Medical School, University of São Paulo, Ribeirão Preto, Brazil; ^2^Department of Biochemistry and Immunology, Ribeirão Preto Medical School, University of São Paulo, Ribeirão Preto, Brazil; ^3^Cell and Molecular Biology and Pathogenic Bioagents, Ribeirão Preto Medical School, University of São Paulo, Ribeirão Preto, Brazil; ^4^Department of Experimental Psychology, Institute of Psychology, University of São Paulo, São Paulo, Brazil

**Keywords:** type 1 diabetes, endothelial dysfunction, NLRP3 inflammasome, mitochondrial DNA, inflammation, reactive oxygen species

## Abstract

**Background:** NLRP3 inflammasome activation in response to several signals, including mitochondrial DNA (mDNA), regulates inflammatory responses by caspase-1 activation and interleukin-1β (IL-1β) release. Circulating mDNA is linked to micro and macrovascular complications in diabetes. However, a role for mDNA in endothelial dysfunction is not clear. We tested the hypothesis that mDNA contributes to diabetes-associated endothelial dysfunction and vascular inflammation via NLRP3 activation.

**Methods:** Vascular reactivity, reactive oxygen species (ROS) generation, calcium (Ca^2+^) influx and caspase-1 and IL-1β activation were determined in mesenteric resistance arteries from normoglicemic and streptozotocin-induced diabetic C57BL/6 and NLRP3 knockout (*Nlrp3^–/–^*) mice. Endothelial cells and mesenteric arteries were stimulated with mDNA from control (cmDNA) and diabetic (dmDNA) mice.

**Results:** Diabetes reduced endothelium-dependent vasodilation and increased vascular ROS generation and caspase-1 and IL-1β activation in C57BL/6, but not in *Nlrp3^–/–^* mice. Diabetes increased pancreatic cytosolic mDNA. dmDNA decreased endothelium-dependent vasodilation. In endothelial cells, dmDNA activated NLRP3 via mitochondrial ROS and Ca^2+^ influx. Patients with type 1 diabetes exhibited increased circulating mDNA as well as caspase-1 and IL-1β activation.

**Conclusion:** dmDNA activates endothelial NLRP3 inflammasome by mechanisms that involve Ca^2+^ influx and mitochondrial ROS generation. NLRP3 deficiency prevents diabetes-associated vascular inflammatory damage and endothelial dysfunction. Our study highlights the importance of NLRP3 inflammasome in diabetes-associated vascular dysfunction, which is key to diabetic complications.

## Introduction

The NLRP3 inflammasome is a multi-protein complex present in cells of the adaptive and innate immune system. NLRP3 regulates inflammatory responses by oligomerization and recruitment of the apoptosis-associated speck-like protein containing a caspase recruit domain (ASC) and pro-caspase-1, causing auto-cleavage and activation of caspase-1, which, in turn, cleaves pro-IL-1β and pro-IL-18 into mature cytokines (Martinon et al., 2002). Mature IL-1β, through the IL-1 receptor (IL-1R), activates various intracellular signaling cascades that trigger transcription of other pro-inflammatory cytokines, adhesion molecules, chemokines and pro-inflammatory enzymes (Palomo et al., 2015). In addition to its role on inflammation, IL-1β is involved in endothelial dysfunction, a hallmark of several cardiovascular and metabolic diseases, including diabetes (Rizzoni et al., 2001; Beckman et al., 2003). IL-1β increases the activity of NADPH oxidase, a key enzyme in oxidative stress-associated conditions, leading to reduced endothelium-dependent vasodilation (Vallejo et al., 2014).

Increased availability of reactive oxygen species (ROS) is a very important mechanism that triggers endothelial dysfunction, being involved in the onset of many diseases. Increased ROS is also a classical mechanism that induces NLRP3 inflammasome activation. Many other stimuli, such as lysosomal damage, potassium (K^+^) efflux, calcium (Ca^2+^) influx, and DAMPs (damage-associated molecular patterns) also induce NLRP3 oligomerization (Brough et al., 2003; Petrilli et al., 2007; Chu et al., 2009; Zhou et al., 2010). Mitochondrial DAMPS, i.e., mitochondrial DNA (mDNA), particularly cytosolic oxidized mDNA, activate the NLRP3 inflammasome and, consequently, increase IL-1β release, as demonstrated in murine macrophages (Shimada et al., 2012).

Increased levels of circulating mDNA were reported in diabetic subjects with microvascular complications, such as retinopathy and nephropathy (Czajka et al., 2015; Malik et al., 2015). In addition, NLRP3 inflammasome activation by mDNA plays a major role in pathogenic cellular immune responses mediated by T lymphocytes during type 1 diabetes development, contributing to damage of insulin-producing β cells, as we recently demonstrated (Carlos et al., 2017).

Although inflammation plays a key role in type 1 diabetes development, few experimental and clinical studies have evaluated the involvement of NLRP3 activation on vascular inflammatory processes and its repercussion on endothelial function. Therefore, this study investigated the functional role of NLRP3 inflammasome, and related signaling pathways, in the development of type 1 diabetes-associated endothelial dysfunction. We tested the hypotheses that mDNA promotes NLRP3 activation in endothelial cells contributing to endothelial dysfunction and that the genetic deficiency of NLRP3 receptor attenuates vascular dysfunction and inflammatory response observed in streptozotocin-induced type 1 diabetes. Considering the high incidence of diabetes, especially its complications, it is important to understand the immunological and pathophysiological mechanisms related to the disease in order to uncover new therapeutic targets for diabetes prevention and treatment.

## Materials and Methods

### Animals

All experimental protocols were performed in accordance with the ARRIVE Guidelines (Animal Research: Reporting of *in vivo* Experiments) and approved by the Ethics Committee on Animal Research of the Ribeirão Preto Medical School – University of São Paulo, Ribeirão Preto, Brazil (protocol no. 26/2015).

Male, 8 to 10 week-old C57BL/6 wild-type (WT) and NLRP3 receptor knockout (*Nlrp3*^–^*^/^*^–^) mice were obtained from the Isogenic Breeding Unit of the Ribeirão Preto Medical School, University of São Paulo, Ribeirão Preto, Brazil. The animals were housed in the animal facility of the Pharmacology Department, Ribeirão Preto Medical School, on 12-h light/dark cycles under controlled temperature (22 ± 1°C) with *ad libitum* access to food and water. After a 1-week acclimatization period, mice were randomly divided into non-diabetic and diabetic groups.

### Induction of Diabetes by Multiple Low Doses of Streptozotocin (MLD-STZ)

Mice were given daily intraperitoneal injections of 40 mg/kg of streptozotocin (Sigma-Aldrich^®^, St. Louis, Missouri, United States) dissolved in 0.1 M sodium citrate (pH 4.5) for five consecutive days. Blood glucose levels, body weight, and diabetes incidence were monitored weekly. Mice were considered diabetic when glucose levels were ≥230 mg/dl after two consecutive determinations under non-fasting conditions. The animals were submitted to experimental protocols 30 days after induction of diabetes. Body weight, blood glucose, and insulin levels are shown in Supplementary Table S1.

### Mitochondrial DNA Isolation

Pancreata from non-diabetic and diabetic mice were submitted to protocols for mitochondria isolation. The pancreatic tissue was homogenized in 5 ml of medium [(in mM): HEPES 10, sucrose 250 and EGTA 1] at pH 7.2, centrifuged at 600 *g* for 5 min and the supernatant collected and centrifuged at 2,000 *g* for 10 min. The pellet containing the isolated mitochondria was recovered, resuspended and centrifuged at 12,000 *g* for 10 min at 4°C followed by centrifugation at 100,000 *g* at 4°C for 30 min. The supernatant was used for DNA extraction with the phenol–chloroform–isoamyl alcohol mixture (Sigma-Aldrich^®^, St. Louis, MO, United States). Finally, pancreatic mDNA isolated from control (cmDNA) and diabetic (dmDNA) mice was quantified using an Epoch^TM^ Microplate apparatus (BioTek Instruments^®^, Winooski, VT, United States).

### Vascular Reactivity – Isolated Mesenteric Resistance Arteries

The method described by Mulvany and Halpern (1977) was used. Animals were euthanized in a carbon dioxide (CO_2_) chamber. Segments of second-branch mesenteric arteries (2 mm in length) were mounted in a small vessel myograph (Danish Myo Tech, Model 620M, A/S, Aarhus, Denmark). Arteries were maintained in Krebs Henseleit solution [(in mM) NaCl 130, KCl 4.7, KH_2_PO_4_ 1.18, MgSO_4_ 1.17, NaHCO_3_ 14.9, glucose 5.5, EDTA 0.03, CaCl_2_ 1.6], at 37°C, pH 7.4, and gassed with a mixture of 95% O_2_ and 5% CO_2_.

Mesenteric arteries preparations were set to reach a tension of 13.3 kPa (kilopascal) and remained at rest for 30 min for stabilization. The arteries were stimulated with Krebs solution containing a high concentration of potassium [K^+^ (120 mM)] to evaluate the contractile capacity. After washing and return to the basal tension, arteries were contracted with phenylephrine (10^–6^ M) and stimulated with acetylcholine (10^–5^ M) to determine the presence of a functional endothelium. Arteries exhibiting a vasodilator response to acetylcholine greater than 80% were considered endothelium-intact vessels. The failure of acetylcholine to elicit relaxation of arteries that were subjected to rubbing of the intimal surface was taken as proof of endothelium removal. After washing and another period of stabilization, concentration-response curves to acetylcholine and sodium nitroprusside were performed.

#### Cumulative Concentration-Response Curves

Mesenteric resistance arteries were pre-contracted with phenylephrine (10^–6^ to 3 × 10^–6^ M) and concentration-response curves to sodium nitroprusside (10^–10^ to 3 × 10^–5^ M), acetylcholine (10^–10^ to 3 × 10^–5^ M) in the presence of vehicle, MCC950 (10^–6^ M), a selective NLRP3 inhibitor, Tiron (10^–4^ M), a superoxide anion scavenger; Peg-catalase (200 U/ml), a catalase mimetic; CCCP (10^–6^ M), an uncoupler of the mitochondrial respiratory chain; and cmDNA and dmDNA (1 μg/ml) were carried out.

### Cultured Endothelial Cells – *EA.hy926* (ATCC^®^ CRL-2922^TM^)

*EA.hy926* endothelial cells were cultured in Dulbecco’s modified Eagle’s medium (DMEM) (Sigma-Aldrich^®^, St. Louis, MO, United States), supplemented with 10% of fetal bovine serum (FBS) (Invitrogen^®^, Carlsbad, CA, United States), and antibiotics (penicillin and gentamicin), at 37°C and 5% CO_2_. Cells (1 × 10^6^/well) were stimulated with cmDNA or dmDNA (1 μg/mL), from 30 min to 1 h. To evaluate caspase-1 and IL-1β activation, cells were primed with lipopolysaccharide (LPS, 1 μg/ml) for 24 h (Sigma-Aldrich^®^, St. Louis, MO, United States), prior to stimulation with mDNA.

### Mitochondrial DNA Quantification

DNA was extracted and purified using the QIAamp DNA Blood Mini kit (Qiagen^®^, Hilden, Germany). DNA isolated from pancreas of mice or from human serum was amplified and quantified using real time-polymerase chain reaction (RT-PCR). The RT-PCR results are presented as the inverse of cycle threshold (CT) for gene amplification (McCarthy et al., 2015). The murine primers used were cytochrome b [(Cyt B) forward 5′-ACCTCAAAGCAACGAAGCCT-3′ and reverse 5′-GGTTGGCCTCCAATTCAGGT-3′], cytochrome c [(Cyt C) forward 5′-GACTTGCAACCCTACACGGAT-3′ and reverse 5′-CCGGTTAGACCACCAACTGT-3′], and NADH dehydrogenase subunit 6 (forward 5′-ATTCCACCCCCTCACGACTA-3′ and reverse 5′-TGTCGTTTTGGGTGAGAGCA-3′). The human primers used were cytochrome b [(Cyt B) forward 5′-ATGACCCACCAATCACATGC- 3′ and reverse 5′-ATGCCCCA ATACGCAAAAT-3′], cytochrome c [(Cyt C) forward 5′– ATGA CCCACCAATCACATGC-3′ and reverse 5′– ATCACATGGCTA GGCCGGAG-3′], and NADH dehydrogenase subunit 6 (forward 5′ – ATACCCATGGCCAACCTCCT-3′ and reverse 5′ – GGGCCTTTGCGTAGTTGTAT-3′).

### Western Blotting

Forty to fifty micrograms of proteins extracted from *EA.hy926* endothelial cells or mesenteric arteries from the different experimental groups were directly loaded into sodium dodecyl sulfate (SDS) sample buffer for 10% SDS- polyacrylamide gel electrophoresis. After protein transfer onto a nitrocellulose membrane (*Trans-*Blot Transfer Medium; Bio-Rad, Hercules, CA, United States), membranes were blocked with 5% bovine serum albumin (BSA) in Tris buffer solution containing 0.1% Tween 20 for 1 h and then incubated with antibodies against NLRP3 (1-500, Abcam, ab4207), Caspase-1 (1-500, Novus Biologicals, 14F468), IL-1β (1-500, Santa Cruz Biotechnology, sc-7884), Nox1 (1-1000, Abcam, ab55831), Nox4 (1-2000, Abcam, ab61248), Catalase (1-2000, Cell Signaling, 8841s), SOD-1 (1-3000, Abcam, ab13498), β-Actin (1-3000, Cell Signaling, #4967) or GAPDH (1-20000, Sigma-Aldrich, G9545) overnight at 4°C. Membranes were then incubated with secondary antibodies, according to species cross-reactivity for each primary antibody, for 1 h at room temperature. After the membranes were rinsed, the immunocomplexes were developed using Luminata^TM^ Forte Western HRP Substrate (Millipore^®^, Burlington, MA, United States) and the images captured in a ImageQuant 350 Photodocumentation system (GE Healthcare^®^, Piscata Way, NJ, United States). The images were quantified by the Image J^®^ program and the results were expressed as arbitrary units (AU).

### Lucigenin

Superoxide anion generation was determined in mice mesenteric arteries and *EA.hy926* endothelial cells by a chemiluminescence assay. Mesenteric arteries from non-diabetic and diabetic C57BL/6 and *Nlrp3*^–^*^/^*^–^ mice were placed in glass tubes containing 950 μl HANK’S solution [(in mM): NaCl 120, CaCl_2_ 1.6, KCl 5, MgCl_2_.6 H_2_O 1, NaH_2_PO_4_ 0.5, glucose 10, HEPES 10] and 5 μl of lucigenin (5 μM) for basal luminescence reading. Then, 50 μl of NAD(P)H (100 μM) were added to the tube and superoxide anion generation was quantified using the Line TL Tube Luminometer (Titertek-Berthold^®^, Pforzheim, Germany). Superoxide anion generation was expressed in RLU (relative units of luminescence)/dry weight (g).

mDNA-stimulated endothelial cells were mechanically removed with 100 μl lysis buffer [(mM) KH_2_PO_4_ 20, EGTA 1] containing a protease inhibitor cocktail [aprotinin 1 μg/ml, leupeptin 1 μg/ml, pepstatin 1 μg/ml, phenylmethylsulfonyl fluoride (PMSF 1 mM)], and then transferred to Eppendorf tubes. In a 96-well white plate, 50 μl of sample, 173.75 μl of phosphate buffer [(in mM): KH_2_PO_4_ 50, EGTA 1, Sucrose 150] and 1.25 μl of lucigenin were pipetted in each well. Basal luminescence reading (3 min) was performed, and 25 μl of NAD(P)H (1 mM) was then added and a new reading (after 15 min) was performed. The Orion II Microplate Luminometer (Titertek-Berthold^®^, Pforzheim, Germany) was used. Superoxide anion generation was expressed in RLU/μg protein.

### Amplex Red

Mesenteric arteries from non-diabetic and diabetic C57BL/6 and *Nlrp3*^–^*^/^*^–^ mice were quickly frozen in liquid nitrogen and subsequently pulverized in ice-cold Krebs solution and centrifuged at 2,000 rpm (1 min). Hydrogen peroxide production was evaluated in aliquots of 50 μl of the supernatant, using an Amplex^TM^ Red Hydrogen Peroxide/Peroxidase Assay Kit (Invitrogen^®^, Carlsbad, California, EUA). The fluorescence was measured (530–590 μm) using the FlexStation 3 Multi Mode Microplate Reader (Molecular Devices, Sunnyvale, CA, United States) and the software SoftMax^®^ Pro (Molecular Devices, Sunnyvale, CA, United States). A standard curve for hydrogen peroxide was constructed to determine hydrogen peroxide concentration in the samples. The quantification was corrected by the total protein concentration and the results are expressed in μmol/l.

### Ca^2+^ Influx

Endothelial cells were seeded in black-walled, clear-bottomed 96-well plates (Corning, NY, United States) at a density of 50,000 cells/well in DMEM with 10% FBS and incubated for 24 h at 37°C in a 5% CO_2_. In the following day, cells were incubated with vehicle, cmDNA or dmDNA (1 μg/ml) diluted in DMEM without phenol red for 30 min. The medium was replaced for 100 μL of dye solution (Molecular Devices, Sunnyvale, CA, United States) and plates were incubated for 1 h at room temperature. Transient changes in Ca^2+^ concentration induced by adenosine 5′-triphosphate (ATP) (10^–5^ M) were measured by fluorescence (515–575 nm) using the FlexStation^®^ equipment and SoftMax^®^ Pro software (Molecular Devices, Sunnyvale, CA, United States). ATP-induced responses were determined immediately upon its addition and measured as peak fluorescent intensity minus basal fluorescent intensity and the area under the curve was calculated.

### Detection of 8-OHdG Levels

The 8-OHdG levels in serum and pancreatic DNA were evaluated by ELISA using the HT 8-oxo-dG ELISA Kit II (Trevigen^®^, Gaithersburg, MD, United States). Briefly, 25 μl of serum samples (1:10), DNA extracted from the pancreas (500 μg/ml), and 8-OHdG monoclonal solution were added in a pre-coated 96-well plate and incubated for 1 h at 25°C, and then washed for four times with phosphate buffered saline containing Tween 20 (PBST). Fifty μl of goat anti-mouse IgG-HRP antibody were then added (incubation for 1 h at 25°C). After four washes with PBST, 50 μl of the TACS-Sapphire^TM^ reagent were added in each well. After 15 min at 25°C, 50 μl of hydrochloric acid (0.2 M) were added and 8-OHdG levels were determined (450 μm) using the FlexStation^®^ equipment and SoftMax^®^ Pro software (Molecular Devices, Sunnyvale, California, United States). A standard curve (3.13 to 200 nM) for 8-OHdG was performed following the manufacturer instructions.

### Drugs and Salts

Phenylephrine hydrochloride, acetylcholine chloride, LPS, lucigenin (N,N′-Dimethyl-9,9′-biacridinium dinitrate) and peg-catalase (Catalase-polyethylene glycol) were obtained from Sigma-Aldrich (St. Louis, MO, United States), MCC950 from Avistron^®^ (Bude, Cornwall, United Kingdom), Tiron from Santa Cruz Biotechnology^®^ (San Juan, CA, United States) and all reagents used in RT-PCR and murine primers from Invitrogen^®^ (Carlsbad, CA, United States). Human primers from Sigma-Aldrich^®^, All other salts used were obtained from Merck^®^ (Rio de Janeiro, RJ, Brazil). For cell culture, DMEM was purchased from Sigma^®^; FBS and antibiotics (Penicillin/Streptomycin) from Gibco Thermo Fisher Scientific^®^ (Waltham, MA, United States).

### Patients

Serum samples from 18 patients with type 1 diabetes and 20 age-matched healthy control subjects (non-diabetic) were collected by a specialized professional from the Clinical Laboratory Service of the University of São Paulo Hospital. All procedures were approved by the Research Ethics Committee of the Institute of Psychology of the University of São Paulo (protocol no. 644.869). Clinical and biochemical characteristics are shown in Supplementary Table S2.

### Statistical Analysis

Results are expressed as mean ±standard error of the mean (E.P.M.). Relaxation responses are expressed as the percentage of relaxation in relation to pre-contraction levels induced by phenylephrine. Concentration-effect curves were submitted to non-linear regression analysis using the GraphPad Prism 6.0 program (GraphPad Software^®^, La Jolla, CA, United States). Agonist potency and maximal response are expressed as pD_2_ [negative log of the molar concentration that produces 50% of maximal response (-log EC_50_)] and Rmax (maximal effect produced by the agonist), respectively.

Statistical analyses were performed by one-way or two-way ANOVA followed by Tukey multiple comparisons post-test for repeated measurements or by Student’s *t*-test for unpaired data. The minimum acceptable level of significance was *P* < 0.05.

## Results

### Type 1 Diabetes Increases Vascular NLRP3 Inflammasome Activation

**FIGURE 1:**
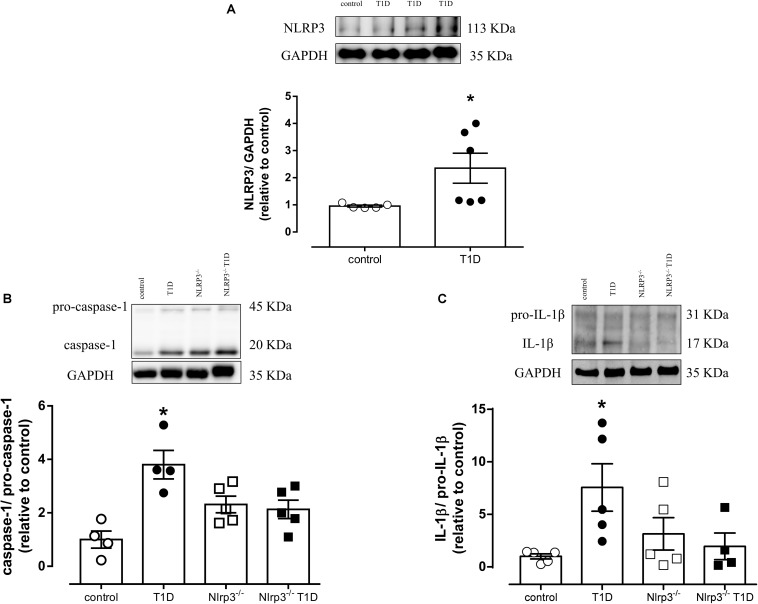
On top, representative images of western blot and, at the bottom, densitometric analysis of protein expression of **(A)** NLRP3, **(B)** caspase-1 and pro-caspase-1 and **(C)** IL-1β and pro-IL-1β in mesenteric arteries from non-diabetic (control, white circles) or diabetic (T1D, black circles) C57BL/6 mice and non-diabetic (*Nlrp3*^–^*^/^*^–^, white squares) or diabetic (*Nlrp3*^–^*^/^*^–^ T1D, black squares) *Nlrp3*^–^*^/^*^–^ mice. Each column represents the mean ±SEM. ^∗^*P* < 0.05 vs. control. *N* = 4–6, Student’s *t*-test **(A)** and two-way ANOVA followed by a Tukey’s *post hoc* test **(B,C)**.

Vascular expression of NLRP3 [Arbitrary Units (AU), T1D = 3.8 ± 0.1 vs. control = 0.9 ± 0.2; *P* < 0.05] (Figure 1A), and caspase-1 [AU, T1D = 3.6 ± 0.5 vs. control = 1.0 ± 0.3; *P* < 0.05] (Figure 1B) as well as IL-1β activation [AU, T1D = 5.5 ± 2.2 vs. control = 1.4 ± 0.2; *P* < 0.05] (Figure 1C) were increased in diabetic WT mice, in comparison to control mice. Diabetic *Nlrp3*^–^*^/^*^–^ mice did not exhibit changes in activation of caspase-1 or IL-1β (Figures 1B,C).

### NLRP3 Activation Contributes to ROS-Induced Endothelial Dysfunction in Type 1 Diabetes

**FIGURE 2:**
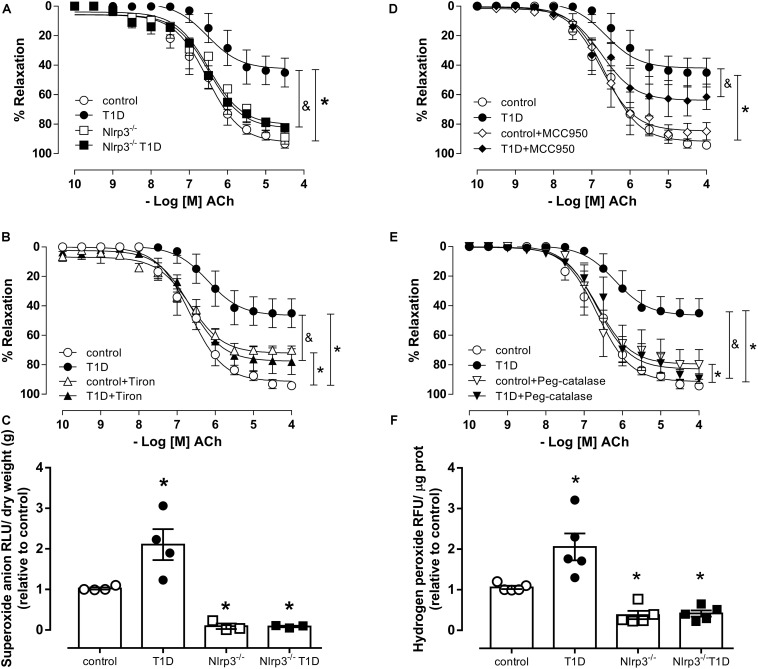
Cumulative concentration-response curves to Acetylcholine (ACh) **(A)**, endothelium-dependent vasodilator, in resistance mesenteric arteries from C57BL/6 non-diabetic (control, white circles) or diabetic (T1D, black circles) and *Nlrp3*^–^*^/^*^–^ non-diabetic (*Nlrp3*^–^*^/^*^–^, white squares) or diabetic (*Nlrp3*^–^*^/^*^–^ T1D, black squares) mice in the presence of **(B)** vehicle (white diamonds) and a NLRP3 inhibitor (MCC950, black diamonds), **(C)** vehicle (white triangles) and superoxide scavenger Tiron (black triangles), and **(D)** vehicle (white triangles down) and catalase mimetic peg-catalase (black triangles down). Generation of **(E)** superoxide anion and **(F)** hydrogen peroxide in mesenteric bed from non-diabetic (control, white circles) or diabetic (T1D, black circles) C57BL/6 mice and non-diabetic (*Nlrp3*^–^*^/^*^–^, white squares) or diabetic (*Nlrp3*^–^*^/^*^–^ T1D, black squares) *Nlrp3*^–^*^/^*^–^ mice. Each point/column represents the mean ±SEM. ^∗^*P* < 0.05 vs. control, ^&^*P* < 0.05 vs. T1D. *N* = 3–7, two-way ANOVA followed by Tukey’s *post hoc* test **(A–F)**.

Endothelium-denuded arteries from control and T1D mice exhibited similar relaxation responses to sodium nitroprusside (Supplementary Figure S1). Mesenteric arteries from T1D mice exhibited decreased endothelium-dependent relaxation (pD_2_, T1D = 6.2 ± 0.2 vs. control = 6.6 ± 0.1; Rmax, T1D = 47.2 ± 4.9 vs. control = 90.2 ± 0.1; *P* < 0.05) (Figure 2A). NLRP3 deficiency prevented diabetes-induced decreased endothelium-dependent relaxation in mesenteric arteries (pD_2_, *Nlrp3*^–^*^/^*^–^ T1D = 6.6 ± 0.1 vs. T1D = 6.2 ± 0.2; Rmax, *Nlrp3*^–^*^/^*^–^ T1D = 80.8 ± 3.3 vs. T1D = 47.2 ± 4.9; *P* < 0.05) (Figure 2A). Reduced vasodilation in WT diabetic mice was partially reverted by MCC950, a selective NLRP3 inhibitor (pD_2_, T1D+MCC950 = 6.9 ± 0.2 vs. T1D = 6.2 ± 0.2; Rmax, T1D+MCC950 = 62.5 ± 3.8 vs. T1D = 46.6 ± 4.1; *P* < 0.05) (Figure 2B), and completely reverted by the superoxide anion scavenger Tiron (pD_2_, T1D+Tiron = 6.6 ± 0.1 vs. T1D = 6.2 ± 0.2; Rmax, T1D+Tiron = 78.4 ± 3.1 vs. T1D = 46.6 ± 4.1; *P* < 0.05) (Figure 2C) and by peg-catalase (pD_2_, T1D+Peg-catalase = 6.4 ± 0.1 vs. T1D = 6.2 ± 0.2; Rmax, T1D+Peg-catalase = 86.4 ± 2.8 vs. T1D = 46.6 ± 4.1; *P* < 0.05) (Figure 2D). MCC950 treatment did not affect ACh responses in arteries from control mice (pD_2_, control+MCC950 = 6.7 ± 0.1 vs. control = 6.6 ± 0.1; Rmax, control+MCC950 = 85.6 ± 6.7 vs. control = 91.3 ± 2.6; *P* > 0.05) (Figure 2B), whereas Tiron (pD_2_, control+Tiron = 6.8 ± 0.1 vs. control = 6.6 ± 0.1; Rmax, control+Tiron = 70.6 ± 1.5 vs. control = 91.3 ± 2.6; *P* > 0.05) (Figure 2C) and Peg-catalase (pD_2_, control+Peg-catalase = 6.8 ± 0.2 vs. control = 6.6 ± 0.1; Rmax, control+Peg-catalase = 78.0 ± 4.4 vs. control = 91.3 ± 2.6; *P* > 0.05) (Figure 2D) produced small decreases in ACh relaxation.

In addition, diabetes significantly increased superoxide anion generation (AU, T1D = 2.1 ± 0.4 vs. control = 1.0 ± 0.1; *P* < 0.05) (Figure 2E) as well as hydrogen peroxide generation (AU, T1D = 2.0 ± 0.4 vs. control = 1.0 ± 0.1; *P* < 0.05) (Figure 2F) in mesenteric arteries from C57BL/6 mice, which was not observed in mesenteric arteries from diabetic *Nlrp3*^–^*^/^*^–^ mice.

### NLRP3 Deficiency Decreases Nox4 Expression and Prevents Increased SOD-1 Expression

**FIGURE 3:**
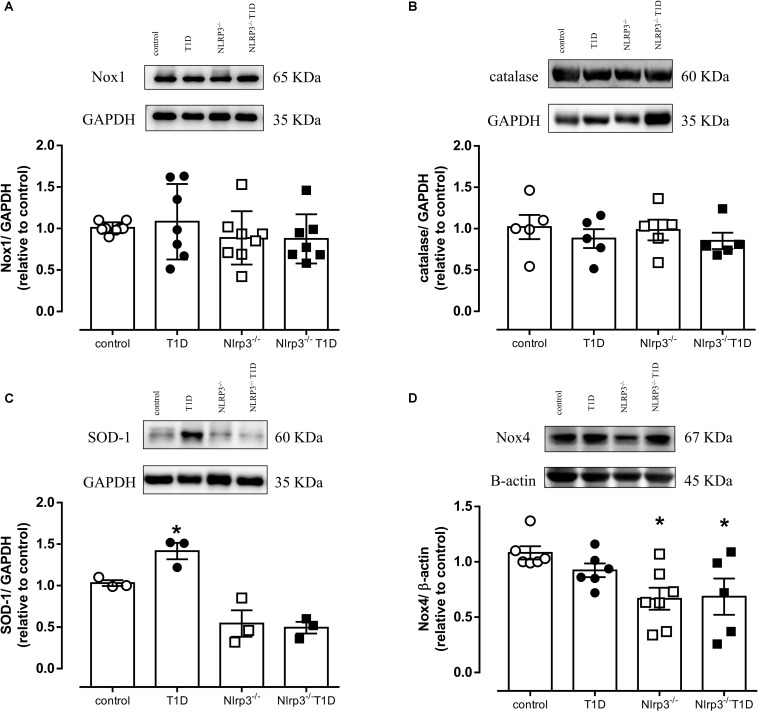
On top, representative images of western blot and, at the bottom, densitometric analysis of the vascular expression of **(A)** Nox1, **(B)** Catalase, **(C)** SOD-1 and **(D)** Nox4 in mesenteric arteries from C57BL/6 non-diabetic (control, white circles) or diabetic (T1D, black circles) and *Nlrp3*^–^*^/^*^–^ non-diabetic (*Nlrp3*^–^*^/^*^–^, white squares) or diabetic (*Nlrp3*^–^*^/^*^–^ T1D, black squares) mice. Each column represents the mean ±SEM. ^∗^*P* < 0.05 vs. respective control. *N* = 3–7, two-way ANOVA followed by Tukey’s *post hoc* test **(A–D)**.

Diabetes did not change expression of Nox1 or catalase (Figures 3A,B) in mesenteric arteries. However, diabetes increased SOD-1 expression in arteries from WT diabetic mice (AU, T1D = 1.4 ± 0.1 vs. control = 1.0 ± 0.1; *P* < 0.05), which was not observed in arteries from *Nlrp3*^–^*^/^*^–^ mice (Figure 3C). In addition, *Nlrp3*^–^*^/^*^–^ mice exhibited reduced vascular Nox4 expression when compared to their respective counterpart C57BL/6 mice (AU, *Nlrp3*^–^*^/^*^–^ = 0.7 ± 0.1 vs. control = 1.1 ± 0.1; *Nlrp3*^–^*^/^*^–^ T1D = 0.7 ± 0.2 vs. T1D = 0.9 ± 0.1; *P* < 0.05) (Figure 3D).

### Type 1 Diabetes Increases Cytosolic mDNA in Pancreatic Cells and dmDNA Promotes Endothelial Dysfunction by Mitochondrial Superoxide Anion Generation

**FIGURE 4:**
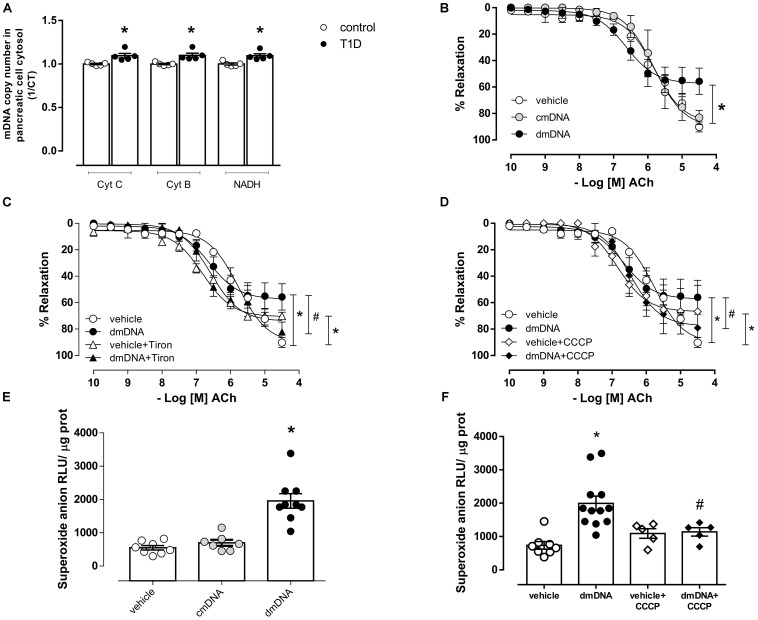
**(A)** Quantification of mitochondrial DNA genes cytochrome C (Cyt C), cytochrome B (Cyt B) and NADH dehydrogenase (NADH) in the cytosolic fraction of pancreatic cells from non-diabetic (control, white circles) or diabetic (T1D, black circles) C57BL/6 mice. Gene expression was determined by RT-PCR. Each bar represents the mean ±SEM. ^∗^*P* < 0.05 vs. control, *N* = 3–5. Cumulative concentration-response curves to Acetylcholine (ACh), endothelium-dependent vasodilator, in resistance mesenteric arteries from C57BL/6 mice incubated with **(B)** vehicle (white circles), mitochondrial DNA from control (cmDNA, gray circles) or diabetic (dmDNA, black circles) mice. Acetylcholine responses in mesenteric arteries incubated with vehicle or dmDNA were also performed in the presence of **(C)** Tiron [vehicle+Tiron (white triangles) or dmDNA+Tiron (black triangles)] and **(D)** CCCP [vehicle+CCCP (white diamonds) or dmDNA+CCCP (black diamonds)]. Superoxide anion generation in endothelial cells incubated with **(E)** vehicle (white circles), cmDNA (gray circles) or dmDNA (black circles) and in the presence of **(F)** CCCP [vehicle+CCCP (white diamonds) or dmDNA+CCCP (black diamonds)]. Each point/column represents the mean ±SEM. ^∗^*P* < 0.05 vs. vehicle, ^#^*P* < 0.05 vs. dmDNA. *N* = 5–12, Student’s *t*-test **(A)**, one-way ANOVA **(B–F)**.

Diabetes increased cytosolic mDNA release (Figure 4A), represented by increased Cyt C, Cyt B, and NADH expression. dmDNA, but not cmDNA reduced endothelium-dependent relaxation (Rmax, dmDNA = 57.3 ± 3.7 vs. vehicle = 90.5 ± 3.4; *P* < 0.05) (Figure 4B) in mesenteric arteries. dmDNA-induced reduced vasodilation was reversed by Tiron (Rmax, dmDNA+Tiron = 74.0 ± 3.4 vs. dmDNA = 57.3 ± 3.7; *P* < 0.05) (Figure 4C) and by CCCP (Rmax, dmDNA+CCCP = 77.7 ± 3.3 vs. dmDNA = 57.3 ± 3.7; *P* < 0.05) (Figure 4D). dmDNA also increased superoxide anion generation in endothelial cells (RLU/μg prot., dmDNA = 1958 ± 217 vs. vehicle = 550 ± 65; *P* < 0.05) (Figure 4E) and CCCP prevented dmDNA-induced increased superoxide anion generation in endothelial cells (RLU/μg prot., dmDNA+CCCP = 1138 ± 126 vs. dmDNA = 1995 ± 219) (Figure 4F). Tiron and CCCP reduced the maximum relaxation response in mesenteric arteries incubated with vehicle (vehicle+Tiron = 70.1 ± 1 vs. vehicle = 90.5 ± 3.4 and vehicle+CCCP = 66.8 ± 5.7 vs. vehicle = 90.5 ± 3.4).

### NLRP3 Activation Contributes to dmDNA-Induced Endothelial Dysfunction

**FIGURE 5:**
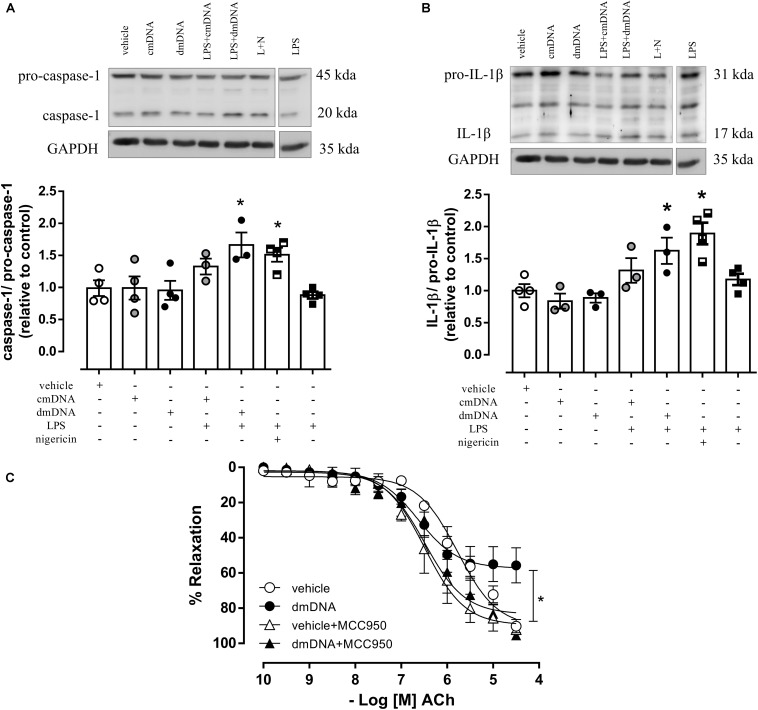
On top, representative images of western blot and, at the bottom, densitometric analysis of the expression of **(A)** caspase-1 and pro-caspase-1 and **(B)** IL-1β and pro-IL-1β in endothelial cells primed with lipopolysaccharide (LPS) and incubated with vehicle (white squares) and mitochondrial DNA from control (cmDNA, white circles) and diabetic (dmDNA, black circles) mice. **(C)** Cumulative concentration-response curves to acetylcholine (ACh), endothelium-dependent vasodilator, in resistance mesenteric arteries from C57BL/6 incubated with vehicle (white circles) or mitochondrial DNA from diabetic mice (dmDNA, black circles) in the presence of vehicle (white triangles) or a NLRP3 inhibitor (MCC950, black triangles). Each point/column represents the mean ±SEM. ^∗^*P* < 0.05 vs. vehicle. *N* = 3–5, one-way ANOVA **(A–C)**.

The incubation of endothelial cells with cmDNA and dmDNA did not activate caspase-1 or IL-1β. However, in cells primed with LPS for 24 h prior to mDNA incubation, dmDNA, but not cmDNA, increased activation of caspase-1 (AU, LPS+dmDNA = 1.5 ± 0.2 vs. vehicle = 0.8 ± 0.1; *P* < 0.05) (Figure 5A) and IL-1β (AU, LPS+dmDNA = 1.6 ± 0.2 vs. vehicle = 1.0 ± 0.1; *P* < 0.05) (Figure 5B). MCC950 prevented dmDNA-induced impairment of endothelium-dependent relaxation in mesenteric arteries (Rmax, dmDNA+MCC950 = 91.8 ± 4.9 vs. dmDNA+vehicle = 57.3 ± 3.8; *P* < 0.05) (Figure 5C). MCC950 did not alter ACh responses in vehicle-treated arteries (Rmax, vehicle+MCC950 = 89.1 ± 4.1 vs. vehicle = 90.5 ± 3.4; *P* > 0.05).

### Ca^2+^ Influx-Induced Mitochondrial ROS Generation by dmDNA Activates Endothelial Cell NLRP3 Inflammasome

**FIGURE 6:**
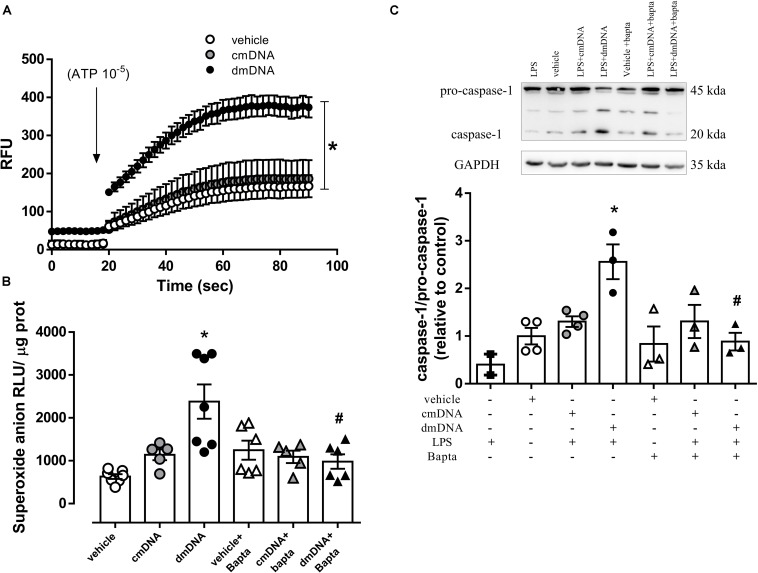
**(A)** Ca^2+^ influx and **(B)** superoxide anion generation in endothelial cells incubated with vehicle (white circles), mitochondrial DNA from control (cmDNA, gray circles) or diabetic (dmDNA, black circles) mice in the presence of vehicle (white triangles) or a Ca^2+^ chelator (Bapta, gray and black triangles). On top, representative images of western blot and, at the bottom, densitometric analysis of the expression of **(C)** caspase-1 and pro-caspase-1 in endothelial cells primed with lipopolysaccharide (LPS) and incubated with vehicle (white squares), mitochondrial DNA from control (cmDNA, gray circles) or diabetic (dmDNA, black circles) mice in the presence of vehicle (white triangles) or a Ca^2+^ chelator (Bapta, gray and black triangles). Each point/column represents the mean ±SEM. ^∗^*P* < 0.05 vs. vehicle, ^#^*P* < 0.05 vs. dmDNA. *N* = 5–12, one-way ANOVA **(A–C)**.

Incubation of endothelial cells with dmDNA, but not cmDNA increased ATP-stimulated Ca^2+^ influx (RFU, dmDNA = 315.0 ± 19.0 vs. vehicle = 130.0 ± 8.6; *P* < 0.05) (Figure 6A). The presence of a Ca^2+^ chelator, Bapta, prevented the increased dmDNA-induced ROS generation (RFU, dmDNA+Bapta = 980 ± 169 vs. dmDNA = 2380 ± 401; *P* < 0.05) (Figure 6B) and caspase-1 activation (AU, LPS+dmDNA+Bapta = 0.9 ± 0.2 vs. LPS+dmDNA = 2.6 ± 0.4; *P* < 0.05) in endothelial cells (Figure 6C).

### Type 1 Diabetes Increases Circulating mDNA and Serum NLRP3 Inflammasome Activation

**FIGURE 7:**
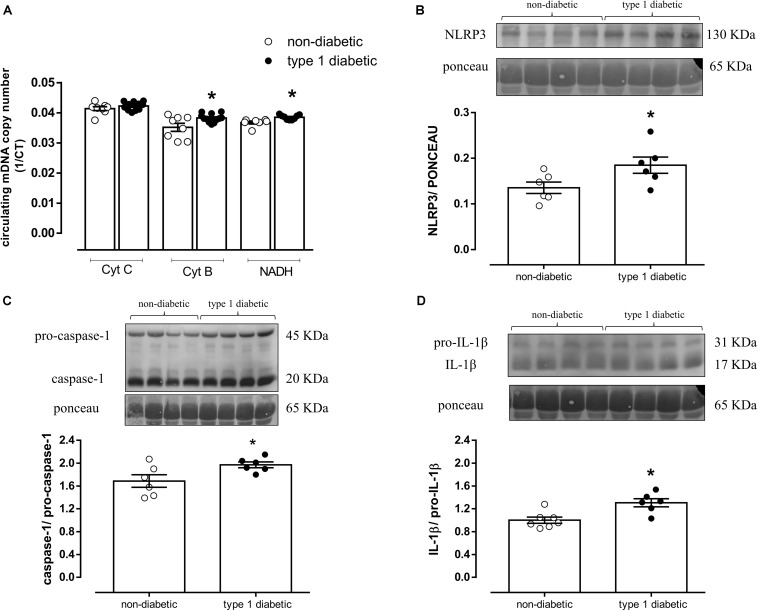
**(A)** Quantification of mitochondrial DNA genes cytochrome C (Cyt C), cytochrome B (Cyt B) and NADH dehydrogenase (NADH). On top, representative images of western blot and, at the bottom, densitometric analysis of the expression of **(B)** NLRP3, **(C)** caspase-1 and pro-caspase-1, and **(D)** IL-1β and pro-IL-1β in the serum from non-diabetic subjects (white circles) and patients with type 1 diabetes (black circles). Each column represents the mean ±SEM. ^∗^*P* < 0.05 vs. non-diabetic. *N* = 5–12, Student’s *t*-test **(A–D)**.

In humans, type 1 diabetes increased circulating mDNA levels represented by increased CytB and NADH expression (Figure 7A). Diabetic patients also exhibited increased NLRP3 expression (AU, Type 1 diabetic = 0.18 ± 0.1 vs. Non-diabetic = 0.13 ± 0.1; *P* < 0.05) (Figure 7B), caspase-1 (AU, Type 1 diabetic = 2.0 ± 0.1 vs. Non-diabetic = 1.7 ± 0.1; *P* < 0.05) (Figure 7C) and IL-1β activation (AU, Type 1 diabetic = 1.3 ± 0.1 vs. Non-diabetic = 1.0 ± 0.1; *P* < 0.05) (Figure 7D). Clinical and biochemical characteristics of control (non-diabetic) subjects and diabetic patients are shown in the (Supplementary Table S2).

### Type 1 Diabetes Does Not Change 8-OHdG Levels

Diabetes did not change 8-OHdG levels in serum DNA or mouse pancreatic mDNA (Supplementary Figure S2).

## Discussion

The present study shows that *Nlrp3*^–^*^/^*^–^ mice are protected from diabetes-associated inflammatory vascular damage and endothelial dysfunction. Mitochondrial DNA is key for NLRP3 inflammasome activation in endothelial cells through increased Ca^2+^ influx and mitochondrial ROS generation.

One of the most important findings of this study is the association between NLRP3 and vascular dysfunction in type 1 diabetes. A link between NLRP3 and endothelial (dys)function has been previously suggested in other studies (15–17). In obese rats, vascular dysfunction, represented by impaired endothelium-dependent relaxation, is associated with increased vascular expression of inflammasome markers such as NLRP3, caspase-1 and IL-1β (Liu et al., 2015). Mice with Kawasaki’s disease, an inflammatory disease model, present impaired endothelium-dependent vasodilation accompanied by increased caspase-1, IL-1β, and VCAM-1 expression (Chen et al., 2015). In addition, NLRP3 deficiency protects endothelial function in hypercholesterolemic mice by reducing vascular superoxide anion generation and increasing eNOS activity (Zhang et al., 2015). *In vitro* experiments also show a modulatory role of NLRP3 on endothelial function since the silencing of NLRP3 gene prevents caspase-1 and IL-1β activation in endothelial cells stimulated with cell wall fragments of *Lactobacillus casei* (Chen et al., 2015). Furthermore, the expression of NLRP3 inflammasome components is increased in brain areas that control blood pressure in spontaneously hypertensive rats and is linked to increased vascular damage and high blood pressure (Avolio et al., 2018).

Reactive oxygen species modulate endothelial function and IL-1β is an important stimulus for ROS production. IL-1β increases ROS generation in human umbilical vein endothelial cells (HUVECs) (Toniolo et al., 2015) and in vascular smooth muscle cells (VSMC) from human coronary arteries (Kaur et al., 2004) by mechanisms that involve increased NADPH oxidase activation. NADPH oxidase is one of the main sources for ROS generation in different organs, including the vasculature (Hansen et al., 2018). Furthermore, inhibition of IL-1β using anakinra, a recombinant human interleukin-1 receptor antagonist, protects endothelial function and reduces ROS generation in mesenteric arteries of diabetic rats (Vallejo et al., 2014). It is important to mention that not only IL-1β induces ROS generation, but ROS also induce IL-1β production. Cultured astrocytes from BALB/C mice stimulated with high glucose concentrations exhibit increased IL-1β, TNF-α, IL-6, and IL-4 gene expression, which is abrogated by a ROS scavenger (Wang et al., 2012).

In the present study, mesenteric arteries from *Nlrp3*^–^*^/^*^–^ mice with type 1 diabetes displayed decreased expression of Nox4 and reduced ROS levels. Other studies support an interaction between NLRP3 inflammasome and Nox expression and activity. Nox4 inhibition prevents ROS generation and decreases protein expression of NLRP3, caspase-1 and IL-1β in endothelial cells stimulated with high glucose (Wang et al., 2017). Decreased NLRP3 inflammasome activation associated with reduced Nox4 expression and ROS generation was also reported in rat cardioblasts stimulated with TNF-α and submitted to hypoxia (Chen et al., 2018). In addition, Nox4 silencing in rat aortic VSMC prevents IL-1β-induced ROS generation (Ginnan et al., 2013). Treatment of mice exhibiting LPS-induced hyperalgesia with a NLRP3 inhibitor, MCC950, decreases the expression of Nox2 subunits, gp91^phox^ and p47^phox^ in the brain, heart and lungs (Dolunay et al., 2017). These studies corroborate a link between Nox4 and NLRP3 inflammasome.

NLRP3 inflammasome activity is triggered by several stimuli, including mitochondrial DAMPs. The immunostimulatory role of mDNA was first reported by Collins et al. (2004). These authors showed that intra-articular administration of mDNA extracted from human and murine tissues induces inflammatory arthritis in mice, an event associated with increased TNF-α levels (Collins et al., 2004). Moreover, mDNA infusion increases IL-6 and neutrophil migration, causing tissue injury (Zhang et al., 2010). Our findings corroborate an immunostimulatory role of mDNA, since mDNA extracted from the pancreas of diabetic mice stimulates the production of proinflammatory markers in endothelial cells. Potential mechanisms associated with the immunostimulatory effect of mDNA include activation of pattern recognition receptors, such as NOD and Toll receptors, and structural modifications of the DNA. TLR-9 activation is linked to mDNA-induced inflammatory responses. TLR-9 antagonist reduces phosphorylation of MAPKs, such as p38 and p44/42, and IL-8 levels in human neutrophils stimulated with mDNA extracted from various tissues (Zhang et al., 2010). TLR-9 inhibition also decreases IL-8 and TNF-α in mDNA-stimulated cells from humans and mice (Pazmandi et al., 2014). NLRP3 is another receptor associated with mDNA effects. LPS-primed macrophages incubated with mDNA exhibit increased activation of caspase-1 and IL-1β in response to ATP, effects associated with NLRP3 activation (Nakahira et al., 2011). In the present study, cells primed with LPS and incubated with dmDNA exhibited increased activation of caspase-1 and IL-1β, reinforcing a relationship between mDNA and NLRP3.

mDNA oxidation has been associated with structural modifications that increase immunogenesis. Oxidized mDNA potentiates increased pro-inflammatory factors (IL-8 and TNF-α) in plasmocytoid dendritic cells (Pazmandi et al., 2014). One of the most common modifications related to DNA oxidation is represented by increased levels of oxidatively modified guanine bases (8-OHdG) (Kuchino et al., 1987). In diabetes, this modification is reported in the urine, mononuclear cells and skeletal muscle of patients (Dandona et al., 1996; Leinonen et al., 1997; Hinokio et al., 1999). Our results did not show higher circulating or pancreatic 8-OHdG levels in the mDNA. However, other modifications, such as epigenetic alterations may increase mDNA immunogenesis. High glucose concentrations increase mDNA methylation, an epigenetic modification, in retinal endothelial cells, a condition also observed in the retinal microvasculature of human donors with diabetic retinopathy (Mishra and Kowluru, 2015). In diabetes, DNA methylation in several organs is associated with micro and macrovascular complications (Kato and Natarajan, 2014). Mitochondrial DNA methylation is also a potential mechanism for the greater immunogenesis associated with diabetic mitochondrial DNA, which should be investigated in further studies.

Another potential mechanism by which mDNA may activate the NLRP3 inflammasome is mitochondrial ROS generation. Human macrophages incubated with increasing doses of inhibitors of the mitochondrial respiratory chain display increased IL-1β release. This effect is inhibited by NLRP3 and caspase-1 gene deletion in both human and murine macrophages (Zhou et al., 2011). In our study, dmDNA increased mitochondrial ROS, since ROS generation by dmDNA in endothelial cells was attenuated by a mitochondrial antioxidant, CCCP. Similar results were observed in murine macrophages after the incubation with a mitochondrial antioxidant agent, MitoTEMPO (Nakahira et al., 2011), reinforcing the link between mDNA, mitochondrial ROS and NLRP3 inflammasome activation. Thus, mitochondrial ROS generation induced by dmDNA seems to activate NLRP3 inflammasome in endothelial cells.

Ca^2+^ influx is also critical for NLRP3 activation and, consequently, IL-1β release. In LPS-primed and ATP-stimulated macrophages, IL-1β release is prevented by thapsigargin, a Ca^2+^ mobilization agent that inhibits the sarcoplasmic Ca^2+^-ATPase (Murakami et al., 2012). Furthermore, mDNA increases Ca^2+^ influx and NLRP3 activation. LPS-primed and ATP-stimulated murine macrophages incubated with mDNA display greater Ca^2+^ influx and activation of caspase-1 and IL-1β (Nakahira et al., 2011). Likewise, in the present study, we found increased Ca^2+^ influx in endothelial cells incubated with dmDNA.

Considering the immunogenic role of mDNA, the presence of circulating mDNA, i.e., mDNA released into the blood, has been associated with inflammatory mechanisms under several conditions. Post-myocardial infarction patients have increased circulating mDNA levels, accompanied by higher levels of inflammatory cytokines, such as TNF-α and IL-6, compared to healthy individuals (Qin et al., 2017). Patients with breast cancer (Mahmoud et al., 2015), gastric cancer (Fernandes et al., 2014), diabetes and retinopathy (Malik et al., 2015), coronary artery disease (Liu et al., 2016) also have higher levels of circulating mDNA. These results corroborate data from our study showing that patients with type 1 diabetes present an increase in circulating mDNA and the increased release of mDNA from pancreatic cells observed in our study may be related to the apoptotic and necrotic processes triggered in the endocrine pancreas by diabetes, which enables extracellular genetic content extravasation. Overall, this is the first study that demonstrates a relationship between mDNA, vascular NLRP3 inflammasome activation, Ca^2+^ influx-induced ROS generation and endothelial dysfunction in diabetes.

## Conclusion

**FIGURE 8:**
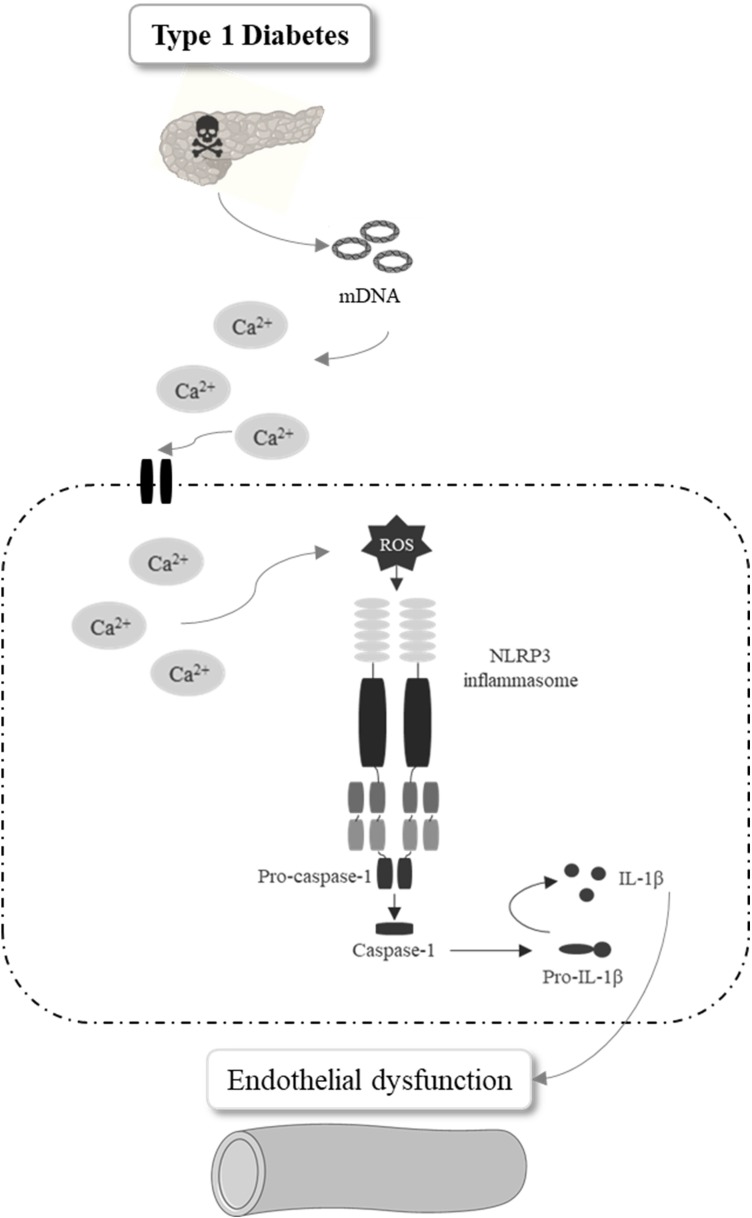
Schematic representation of the conclusion. Pancreatic mitochondrial DNA release in type 1 diabetes increases Ca^2+^ influx and mitochondrial ROS generation in endothelial cells promoting caspase-1 and IL-1 secretion by NLRP3 inflammasome activation, events that contribute to diabetes-associated endothelial dysfunction.

In summary, mDNA contributes to endothelial dysfunction in type 1 diabetes, which is linked to increased inflammatory mediators via activation of the NLRP3 inflammasome in endothelial cells (Figure 8). Pharmacologic inhibition or genetic deletion of the NLRP3 in mice protects from diabetes-associated inflammatory vascular damage and endothelial dysfunction. Our study highlights the importance of NLRP3 inflammasome in diabetes-associated vascular dysfunction, which is key to diabetes-associated complications.

## Data Availability Statement

The raw data supporting the conclusions of this article will be made available by the authors, without undue reservation, to any qualified researcher.

## Ethics Statement

The studies involving human participants were reviewed and approved by the Research Ethics Committee of the Institute of Psychology of the University of São Paulo (protocol no. 644.869). The patients/participants provided their written informed consent to participate in this study. The animal study was reviewed and approved by the Ethics Committee on Animal Research of the Ribeirão Preto Medical School – University of São Paulo, Ribeirão Preto, Brazil (protocol no. 26/2015).

## Author Contributions

CP, NF, CZ, and JFS performed the wet laboratory experiments. CP, DC, and RT designed the study. VG and DV provided the human samples. DZ and JSS provided the knockout mice used in this study. CP wrote the manuscript. DC, DZ, and RT revised its scientific content.

## Conflict of Interest

The authors declare that the research was conducted in the absence of any commercial or financial relationships that could be construed as a potential conflict of interest.
