# Supplementary material for: Mitochondrial DNA Promotes NLRP3 Inflammasome Activation and Contributes to Endothelial Dysfunction and Inflammation in Type 1 Diabetes
Source: Front Physiol. 2020 Jan 17;10:1557. doi: 10.3389/fphys.2019.01557 (PMC6978691; doi:10.3389/fphys.2019.01557)
Supplement: Supplementary file 1 [file Data_Sheet_1.pdf]

Supplementary figure 1: Cumulative concentration-response curves to Sodium Nitroprusside (SNP), endothelium-independent vasodilator, in resistance mesenteric arteries from C57BL/6 non-diabetic (control, white circles) or diabetic (T1D, black circles). Each point represent the mean ± S.E.M.

Supplementary figure 2: 8-OHdG levels in (A) DNA extracted from serum and (B) mitochondrial DNA extracted from the pancreas of C57BL/6 non-diabetic (control, white circles) or diabetic (T1D, black circles) mice. Each point represents the mean ± S.E.M. *, P <0.05 vs. control, N = 5-7.

Supplementary Table 1: Body weight, blood glucose and insulin levels of the experimental groups C57BL/6 and Nlrp3$^{-/-}$ non-diabetic and diabetic

	C57BL/6 non-diabetic **N=6**	C57BL/6 diabetic **N=7**	Nlrp3$^{-/-}$ non-diabetic **N=6**	Nlrp3$^{-/-}$ diabetic **N=8**
Body weight (g)	25.5 ± 0.2	25.3±0.5	25.1±0.7	25.1 ± 0.6
Body glucose (mg/dL)	158 ± 0.4	316 ± 1.2*	168 ±1.5	263 ± 1.4*
Insulin (µl/ml)	32.2 ± 1.6	23.9 ± 1.5*	30.8 ± 4.2	28.5 ± 3.1

Values are in mean ± SEM; N (number); *, P <0.05 vs. C57BL/6 non-diabetic.

Supplementary Table 2: Clinical and biochemical characteristics of healthy controls and patients with type 1 diabetes

**Characteristics**	**Control**	**Patients with type 1 diabetes**
Female (N)	12	11
Male (N)	8	7
Age (years)	26,0 ± 1,6	27,5 ± 1,4
Diabetes Duration (years)	/	14,0 ± 1,6
HbA1c (%)	5,3 ± 0,3<br>5.3 % NGSP = 34 mmol/mol IFCC = 105 mg/dL eAG	8,6 ± 0,4<br>8.6 % NGSP = 70 mmol/mol IFCC = 200 mg/dL eAG

Values are in mean ± SEM; N (number); HbA1c (Glycated hemoglobin); NGPS (National Glycohemoglobin Standardization Program); IFCC (International Federation of Clinical Chemistry); eAG (Estimated average glucose)
