# Supplementary figures and images for: Mitochondrial DNA Promotes NLRP3 Inflammasome Activation and Contributes to Endothelial Dysfunction and Inflammation in Type 1 Diabetes
Source: Front Physiol. 2020 Jan 17;10:1557. doi: 10.3389/fphys.2019.01557 (PMC6978691; doi:10.3389/fphys.2019.01557)

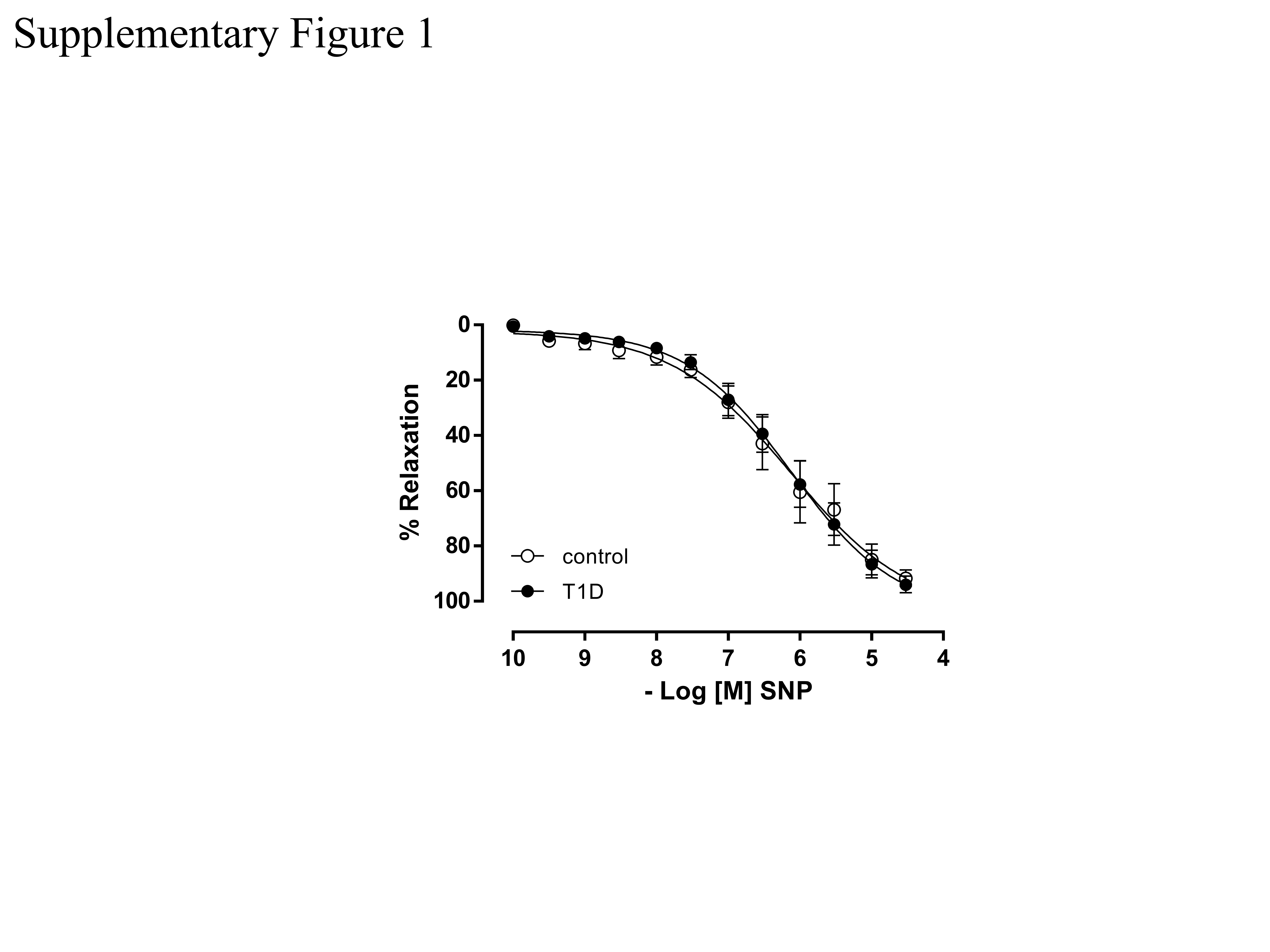

Supplement: Supplementary file 2 [file Image_1.TIF]

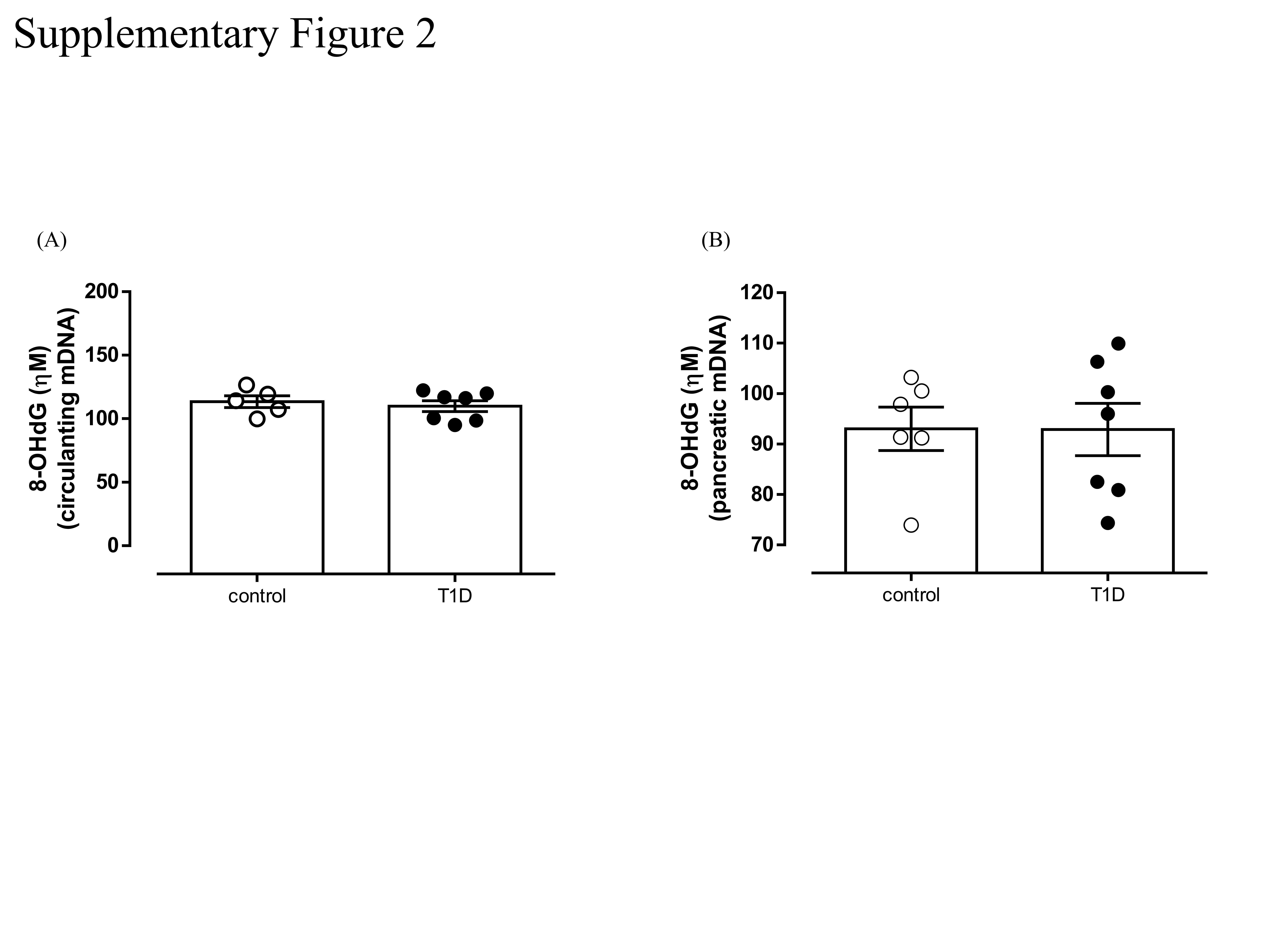

Supplement: Supplementary file 3 [file Image_2.TIF]
